# Dynamic Bowtie Filter for Cone-Beam/Multi-Slice CT

**DOI:** 10.1371/journal.pone.0103054

**Published:** 2014-07-22

**Authors:** Fenglin Liu, Qingsong Yang, Wenxiang Cong, Ge Wang

**Affiliations:** 1 Key Lab of Optoelectronic Technology and System, Engineering Research Center of Industrial Computed Tomography Nondestructive Testing, Ministry of Education, Chongqing University, Chongqing, China; 2 Biomedical Imaging Center, Center for Biotechnology and Interdisciplinary Studies, Department of Biomedical Engineering, Rensselaer Polytechnic Institute, Troy, New York, United States of America; Institute of Automation, Chinese Academy of Sciences, China

## Abstract

A pre-patient attenuator (“bowtie filter” or “bowtie”) is used to modulate an incoming x-ray beam as a function of the angle of the x-ray with respect to a patient to balance the photon flux on a detector array. While the current dynamic bowtie design is focused on fan-beam geometry, in this study we propose a methodology for dynamic bowtie design in multi-slice/cone-beam geometry. The proposed 3D dynamic bowtie is an extension of the 2D prior art. The 3D bowtie consists of a highly attenuating bowtie (HB) filled in with heavy liquid and a weakly attenuating bowtie (WB) immersed in the liquid of the HB. The HB targets a balanced flux distribution on a detector array when no object is in the field of view (FOV). The WB compensates for an object in the FOV, and hence is a scaled-down version of the object. The WB is rotated and translated in synchrony with the source rotation and patient translation so that the overall flux balance is maintained on the detector array. First, the mathematical models of different scanning modes are established for an elliptical water phantom. Then, a numerical simulation study is performed to compare the performance of the scanning modes in the cases of the water phantom and a patient cross-section without any bowtie and with a dynamic bowtie. The dynamic bowtie can equalize the numbers of detected photons in the case of the water phantom. In practical cases, the dynamic bowtie can effectively reduce the dynamic range of detected signals inside the FOV. Furthermore, the WB can be individualized using a 3D printing technique as the gold standard. We have extended the dynamic bowtie concept from 2D to 3D by using highly attenuating liquid and moving a scale-reduced negative copy of an object being scanned. Our methodology can be applied to reduce radiation dose and facilitate photon-counting detection.

## Introduction

X-ray computed tomography (CT) is a corner stone of modern hospitals and clinics, and still under rapid development. Two contemporary topics are radiation dose reduction and multi-energy imaging. These two topics are actually interconnected. Let us first review some background information, which will lead to the main point of this paper on a 3D dynamic bowtie design.

CT radiation dose is a public major concern, especially for children. A British study quantified the cancer risk associated with the use of diagnostic x-rays, arguably causing ∼700 cases of cancer per year in Britain and >5,600 cases in US [1]. Hence, the well-known ALARA (“*As Low As Reasonably Achievable*”) principle has been accepted. It is ideal to send x-ray photons along each path as less as possible.

CT contrast resolution is rather poor in the context of soft tissue imaging. X-ray detection technology has been almost exclusively based on energy-integration. On the other hand, the best photon-counting detectors recognize photons individually and spectrally. Photon-counting detectors can reveal elemental composition and support contrast-enhanced studies through K-edge imaging [2]. However, the dynamic range of the photon-counting detector is rather limited. When the flux on the detector is lower than the maximum count rate, the imaging performance is excellent, but if multiple photons arrive in temporal proximity, the detector may not be able to resolve them as separate events. This loss means spectral distortion [3]. Hence, it is highly desirable to prescribe the attenuated photon flux on a per-ray basis.

Over the past years, CT dose reduction and multi-energy CT have attracted a major attention. For that purpose, optimally balancing an attenuated x-ray flux distribution is a pre-requisite. Up to date, a pre-patient attenuator, which is called a “bowtie filter” or “bowtie”, is already in use. Such a bowtie filter selectively attenuates photons emitted from an x-ray source as a function of the angle of an x-ray [4]. Thus, the bowtie compresses the dynamic range on the detector by increasing attenuation for x-rays further from the iso-center of a field of view (FOV), which typically travel through less tissue. Thus, the bowtie helps improve image quality, since readings of the detectors are substantially equalized, the dynamic range is reduced for more detailed information quantization [5]. Also, by blocking low-energy x-rays, the bowtie also works with an x-ray beam filter to reduce the beam-hardening effect [6]. Furthermore, by blocking radiation to the periphery of a patient where the attenuation path is the shortest, the radiation dose is reduced, so is the scatter-to-primary ratio [7]–[9]. The limitation of the current bowtie filter is that the attenuation profile it produces is fixed and cannot be adaptively changed with the gantry rotation. Although modern CT scanners employ a small number of bowtie filters for different applications, these filters are not personalized and must be fixed for an entire scan.

As a complementary method, an attenuation-based tube current modulation method was proposed to allow dose reduction [10], [11]. This method modulates an incoming x-ray flux as a function of the view angle, instead of the angle of an individual x-ray. Hence, the tube current modulation can be customized on a per-patient basis. However, the problem is that this modulation changes neither the scatter to primary ratio nor the dynamic range for any given view angle.

Because the combination of a traditional bowtie filtration and a tube current modulation cannot meet the sophisticated needs for CT dose reduction and multi-energy imaging, a dynamic bowtie was proposed to smartly filter the radiation emitted towards a patient in synchrony with a data acquisition process. Along this direction, various beam-shaping filters were designed. The effects of the bowtie design on image quality and radiation dose were also reported [12]–[14]. Recently, the feasibility of a flexible piecewise-linear dynamic bowtie was studied [15], in which a set of triangular wedges were used as a basis to approximate any desirable attenuating profile. The initial simulation results showed a major reduction in the dynamic range of raw data. In an earlier study, we proposed a dynamic bowtie design for fan-beam CT, which uses a rotating bowtie filter for control within each fan-beam coupled with a tube current modulation for control across view angles [16]. However, these dynamic bowtie designs are fan-beam geometry oriented, and not intended for moderate or large cone angles.

Inspired by a dynamic beam-shaper report and a digitally controlled beam attenuator utilizing a highly attenuating fluid controlled with pistons [17], [18], in this paper, we focus on a simple, general and easy-to-implement methodology for 3D bowtie design. The proposed 3D dynamic bowtie is an extension of the 2D prior art. The 3D bowtie consists of a highly attenuating bowtie (HB) filled in with heavy liquid and a weakly attenuating bowtie (WB) immersed in the liquid of the HB. The HB targets a balanced flux distribution on a detector array when no object is in the field of view (FOV). The WB compensates for an object in the FOV, and hence is a scaled-down version of the object. The WB is rotated and translated in synchrony with the source rotation and patient translation so that the overall flux balance is maintained on the detector array. In the next section, representative 3D bowties for different imaging modes are analyzed, and the HB contours are derived for fan-beam CT and multi-slice/cone-beam CT including helical CT. In the third section, numerical simulation results are reported to demonstrate the performance of the dynamic bowtie in cone-beam geometry. In the last section, relevant issues are discussed.

## Methodology

When an x-ray beam irradiates an object in cone-beam geometry, the length of each x-ray path varies significantly as a function of the angular position of the ray within a tilted fan-beam, the tilting angle, and the object. If these variations are not effectively compensated, a large dynamic range will be required, or a data overflow problem will be generated in detectors. This is especially problematic for spectral detectors whose counting rates are much slower than the current integrating counterparts. To meet such a challenge, a smart bowtie can be used to optimally shape the x-ray beam so that the expected numbers of photons are equalized across detector channels. The major task of the dynamic bowtie design is to determine the bowtie shape and its dynamics to undo the path length changes during a CT scan. In this following, we first analyze the bowtie filter profile for the central beam plane in fan-beam geometry, and then extend the idea to cone-beam geometry. For convenience, we assume a monochromatic x-ray source without loss of generality.

### Bowtie Filter for Fan-beam CT

**Figure 1:**
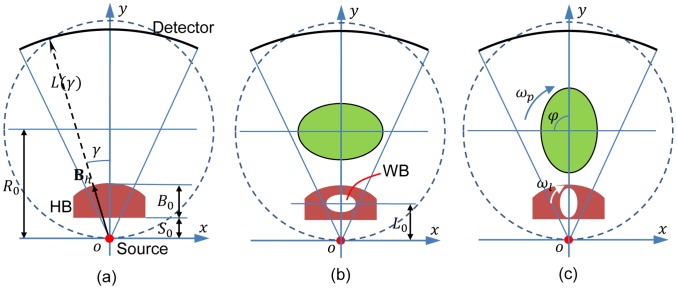
Dynamic bowtie in fan-beam geometry for a balanced flux distribution upon an equiangular detector array. (a) No object in the field of view (FOV) corresponds to a liquid highly attenuating bowtie (HB); (b) an elliptical phantom in the FOV corresponds to the HB containing a weakly attenuating bowtie (WB) that compensates for the attenuation due to the phantom; and (c) the WB is synchronously rotated with the gantry for dynamic compensation (for example, 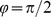).

On the central imaging plane, a multi-slice/cone-beam becomes a fan-beam. We assume a fan-beam geometry with detector cells equi-angularly distributed. An x-ray source is rotated along a circular trajectory of radius 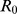. Let 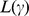 denotes a ray in the fan-beam, where the angle 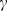 specifies the ray in the fan-beam. The coordinate systems are established in FIG. 1, where 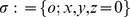 is fixed with the source. We first require that when no object is in the field of view (FOV) data detected are uniform with a bowtie. Then, if we place a high attenuation liquid bowtie (HB) shown as FIG. 1 (a) for uniform detector readings, the profile 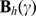 of the bowtie can be expressed in 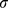 as
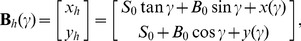
(1)where 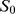 is the distance from the source to the HB, 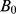 is the attenuation length of the HB. 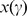 and 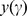 define a bowtie layer to compensate for the inhomogeneous intensity distribution of x-rays from an x-ray source.

Since a cross-section of the human head, chest, and abdomen can be approximated to be elliptical, the bowtie filter can be analytically designed for such objects. As illustrated in FIG. 1 (b), when an elliptical water phantom with a semi-major axis 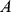 and a semi-minor axis 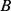 is placed within the fan-beam, the acquired data will vary according to different path lengths. Then, the fan-beam projection 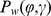 through the homogenous elliptical object with an attenuation coefficient 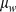 can be derived as [19]

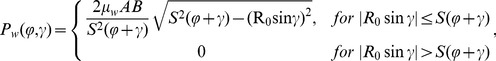
(2)where 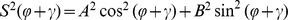.

To make the acquired data uniform, we can insert an elliptical low attenuation chamber (the WB) with a semi-major axis 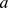 and a semi-minor axis 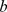 into the HB. When the WB is rotated synchronously with the source, the variation of projection 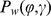 can be perfectly compensated for. For that purpose, we have the net projection expressed as
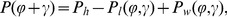
(3)Where 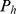 is the full projection only with the HB (without a WB) which yields uniform projection data by design, 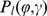 is the differential projection of the high density liquid replaced by the WB. Let 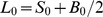, 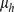 and 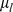 be the attenuation coefficient of the HB and WB respectively. If
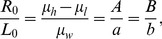
(4)we have
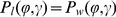
(5)and
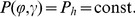
(6)


That is, we can have a constant projection profile during a full scan.

Our proposed dynamic bowtie for fan-beam CT is to have a rotating solid WB in a stationary liquid HB. An earlier dynamic beam shaper reported by Roessl et al. used a circular metal piece with a reduced elliptical patient inside, which is rotated in synchrony with the source to compensate for heterogeneous x-ray path lengths through the patient and a “pre-shaper” to equalize the x-ray flux [17]. Both the methods use a reduced version of the patient for the compensation purpose, and they both perform the flux normalization. The main differences between these methods lie in the following aspects. First, the attenuating materials are different. The key component for our design is the use of highly attenuating liquid, instead of rigid solid. Second, the main functionalities are different. Actually, our method covers the previously reported method as a special case (fan-beam geometry is a special case of cone-beam geometry). It is underlined that the key idea is to introduce a highly attenuating liquid as the background medium so that the dynamic compensation can be realized in cone-beam geometry allowing circular and spiral multi-slice/cone-beam scanning modes.

### Bowtie Filter for Multi-slice/Cone-Beam CT

**Figure 2:**
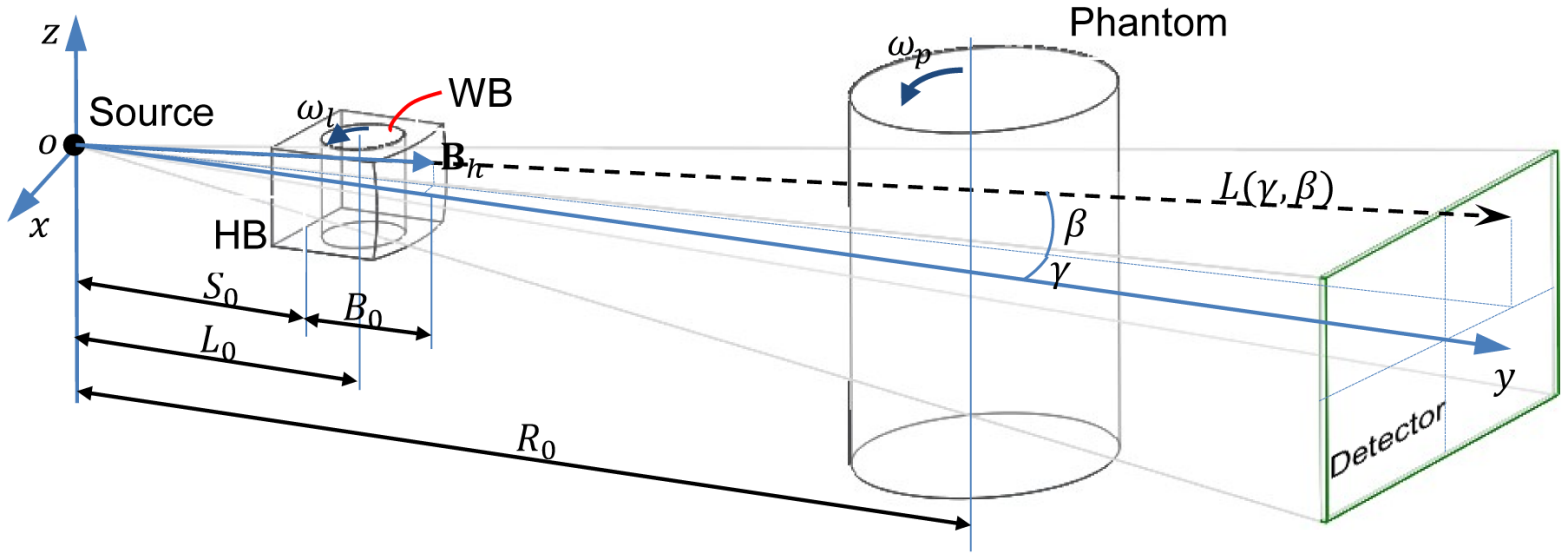
Dynamic bowtie in a cone-beam geometry with a flat panel detector plate.

Now, let us extend the dynamic bowtie design from fan-beam geometry to multi-slice/cone-beam geometry. The coordinate system for cone-beam CT is in FIG. 2, where 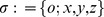 is fixed with the source. Let 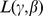 denote a ray within the cone-beam, where 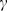 and 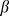 specifies the angle in reference to the two orthogonal central planes respectively. Similar to the fan-beam case, we want to define a surface for the HB to produce uniform detector data, the surface 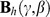 of the HB can be expressed in 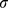 as follows:
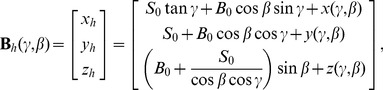
(7)where 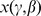, 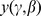, 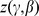 define a bowtie layer for x-ray flux normalization, similar to what we have formulated in the fan-beam case.

Let us use a cylindrical water phantom of an elliptical cross-section with a semi-major axis 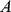 and a semi-minor axis 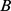 in the cone-beam case, since a patient is quite similar to a cylinder. Then, the acquired multi-slice/cone-beam data will have more variations than in the fan-beam case. Mathematically, the multi-slice/cone-beam projection 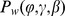 through the homogenous cylindrical phantom with an attenuation coefficient 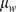 can be obtained as
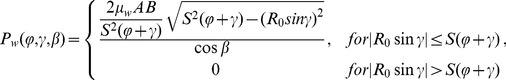
(8)where 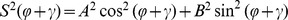.

Just like what we have done in the fan-beam case, to make the acquired data have identical expected values, we can insert a cylindrical WB with a semi-major axis 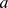 and a semi-minor axis 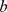 into the HB. When the WB and the source are rotated in synchrony, the variation in 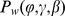 can be appropriately canceled out. Specifically, we have the whole projection expressed as
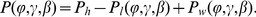
(9)


If we set the parameters according to Eq. (4), we have
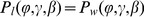
(10)and
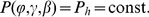
(11)


### Bowtie Filter for Helical CT

**Figure 3:**
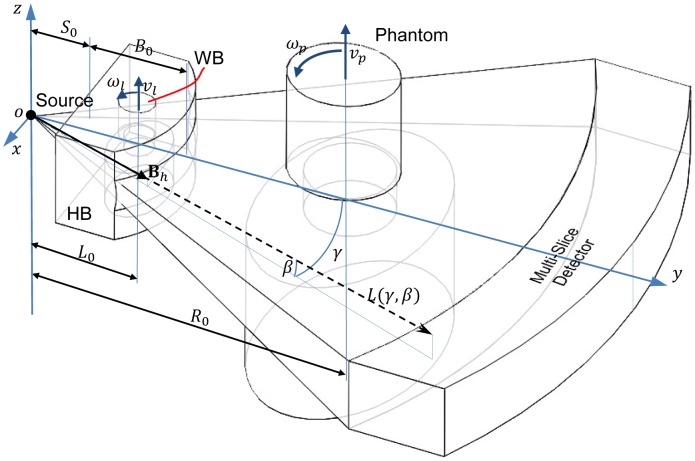
Dynamic bowtie in a spiral multi-slice geometry with a multi-slice detector array.

As illustrated in FIG. 3, we can also handle a helical multi-slice/cone-beam scan as well. The difference between such circular and helical scans will be significant only if a longitudinally non-uniform object is imaged, such as a patient. In this case, the WB should be a miniature of the object to be scanned, and the WB motion must be different from that for circular multi-slice/cone-beam scanning. In addition to the synchronized rotation of the WB and the source, the WB needs to be translated as well in synchrony with the object translation but at a slower speed. Let 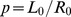, then we have
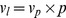
(12)where 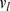 and 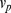 are the translation speeds of the WB and the object respectively.

### Bowtie Design

The dynamic bowtie is a purposely-shaped highly attenuating liquid container with a customized low attenuation bowtie inside that can be moved under precise control in synchrony with both the source rotation and the patient translation. When a bowtie is designed, there are two major factors to be taken into account. The first factor is the selection of the highly attenuating liquid and weakly attenuating material, and the other one is the material to make the container for HB. Based on medical CT applications, our initial choice of the ideal attenuation coefficients for HB and WB are 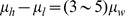, which means the size of the WB is (1/3–1/5) of the object to be scanned. An ideal casing material of the HB should have the same attenuation characteristics as the liquid, while the attenuation coefficient of the WB should be as low as possible. Since air is an ideal WB material with almost zero attenuation, in the following design and simulation, we focus on the WB which is an air chamber with a thin-walled low attenuation container (an interesting point we want to point out is that we can use a 3D printing technique such as pseudo halftone [20] to produce an inhomogeneous weakly attenuating chamber to mimic a patient more realistically).

**Figure 4:**
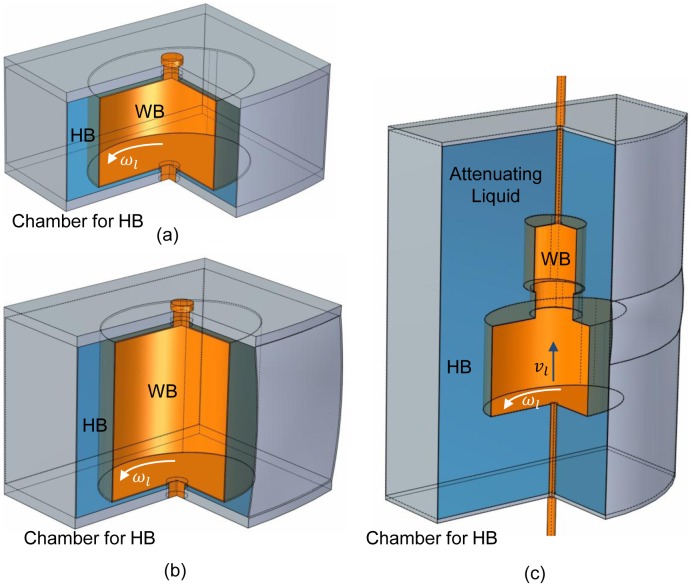
Exemplary designs of the dynamic bowtie without driving components. (a) A dynamic bowtie for fan-beam CT, (b) and (c) for cone-beam CT and spiral multi-slice CT respectively. The attenuating liquid for HB is 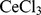 solution. The WB material is air. The containers for HB and WB are made of 0.5 mm thick aluminum and 0.2 mm thick plastic, respectively.

Some exemplary dynamic bowties are shown in FIG. 4, without driving components. In the designs, the highly attenuating liquid is cerous chloride (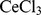) because it is quite soluble, and we can have 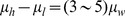 by adjusting its concentration [18]. The HB container material can be 0.5 mm thick aluminum. The WB attenuator is air and its container material can be 0.2 mm thick C-552 air-equivalent plastic [21].

## Numerical Simulation

### Idealized Design

Table 1 lists the parameters used for the design of an exemplary dynamic bowtie. To simulate the dynamic range of detector readings, a mono-energetic x-ray tube was assumed to work at 100 keV, and the numbers of detected photon were the same 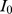 without a bowtie for a blank scan. When the WB is an air chamber with attenuation coefficient 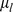, the bowtie was designed with the liquid of attenuation coefficient 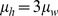, as aforementioned, for a water cylinder of an elliptical cross-section of a semi-major axis 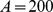 mm and a semi-minor axis 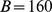 mm.

**Table 1:** Parameters used for the design of a dynamic bowtie.

Parameter	Value
Source trajectory	Full circle
Scan radius ( 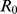 )	57 cm
Source to detector distance (*SDD*)	114 cm
X-ray energy (keV)	100
Number of projections	1160
Number of detector pixels	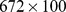
Detector slice thickness ( 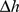 )	2 mm
Detector angular aperture ( 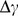 )	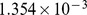 radian
HB container material	Aluminum
HB container thickness ( 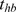 )	0.05 cm
HB container attenuation ( 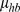 ) [27]	0.460 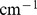
HB liquid attenuator	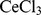 solution
HB liquid attenuation ( 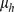 )	3 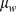
HB attenuation length ( 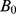 )	14 cm
WB attenuator	Air
WB container material	C-552 air-equivalent plastic
WB container thickness ( 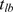 )	0.02 cm
WB container attenuation ( 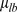 ) [21]	0.112 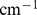
Water attenuation coefficient ( 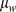 ) [28]	0.171 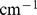
Source to bowtie distance ( 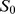 )	12 cm

**Figure 5:**
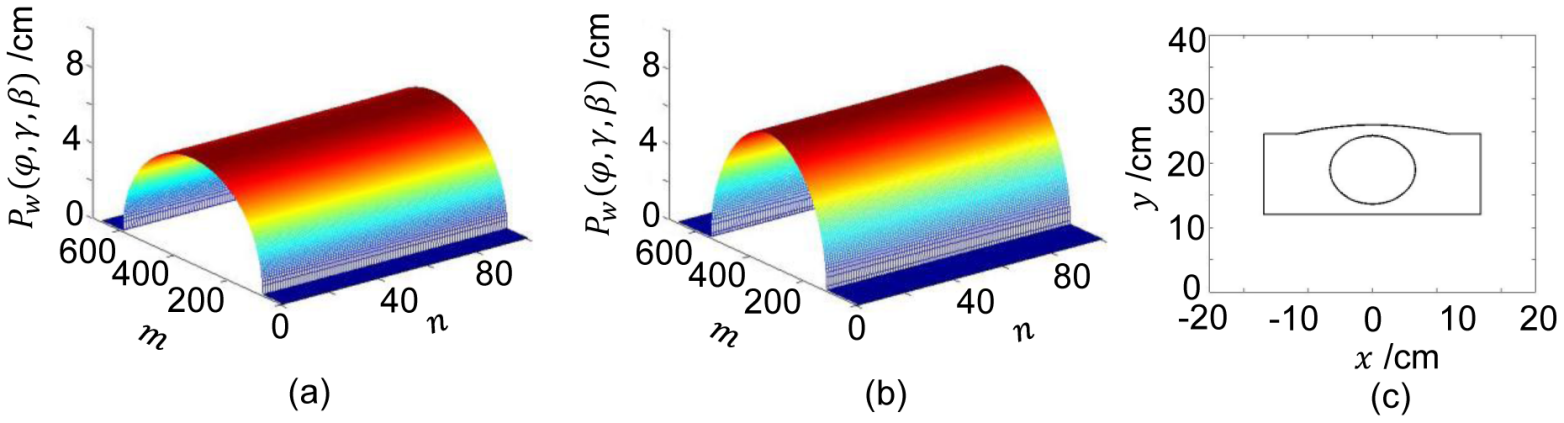
Projections of an elliptical water phantom and the bowtie profile. The projection angle 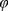 is indexed by the projection number 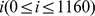, 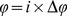, 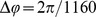. The ray angle 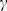 is indexed by the horizontal detector number, 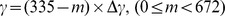. The ray angle 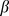 is indexed by the vertical detector number, 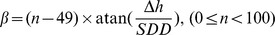. (a)–(b) Surface displays of the sinogram from a water phantom for 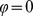 and 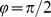 respectively, and (c) the bowtie profile for 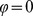 and 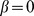.

FIG. 5 (a) shows projection profiles 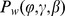 of the water phantom without any bowtie. Setting the projection angle 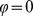 and the fan angle 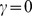, the minimum projection value along the central ray 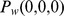 was obtained. Setting 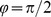 and 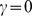, the maximum value along the central ray 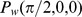 was obtained.

The side of the HB facing the source was assumed flat for convenience. Then, with Eq. (7), the surface of HB 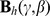 was computed. The WB was rotated angularly around the axis perpendicularly through the bowtie center. By Eq. (4), the WB is an elliptical chamber scaled down from an object by a factor of 1/3 of the object, which means an elliptical cylinder chamber with a semi-major axis 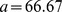 mm and a semi-minor axis 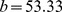 mm. FIG. 5 (c) visualizes the bowtie for 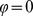 and 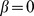.

### Numerical Test

**Figure 6:**
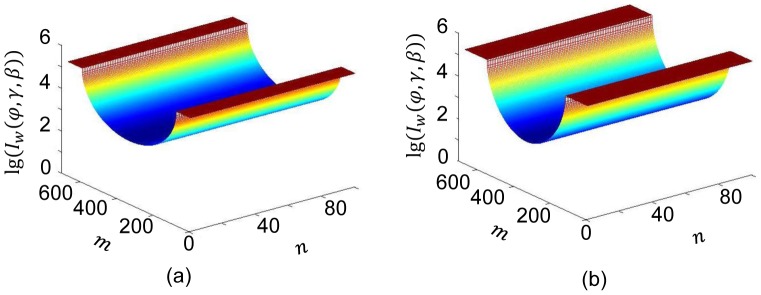
Numbers of detected photons along x-rays through the water phantom without any bowtie (on a log scale). (a) and (b) Surface displays for the numbers of detected photons for 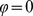 and 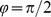 respectively.

First, the numbers of detected photons without the dynamic bowtie were calculated for the water phantom of 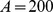 mm and 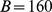 mm over a full scan. By Beer's law,
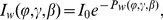
(13)the numbers of projection data were synthesized, as plotted in FIG. 6 for 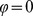 and 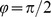 respectively, assuming the number of emitted photons per second 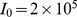 along each ray path.

Second, with the dynamic bowtie for the aforementioned phantom and 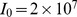 (the increment in the flux is to overcome the attenuation of the bowtie), the numbers of photons were simulated again for each ray path. Practically, the influence of the container materials of the HB and WB should not be ignored. Then, by Eq. (11) we have
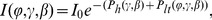
(14)


Where 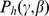 is the projection of the HB, 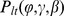 is the projection of the WB container, and
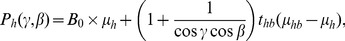
(15)

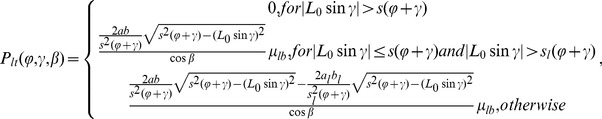
(16)where 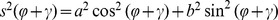, 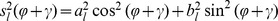, 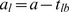, 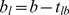.

From Eqs. (14)–(16), when 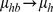, 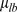 and 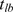 are small enough, we have 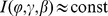, which means that we can effectively regulate the numbers of detected photons along each ray path through the water phantom.

**Figure 7:**
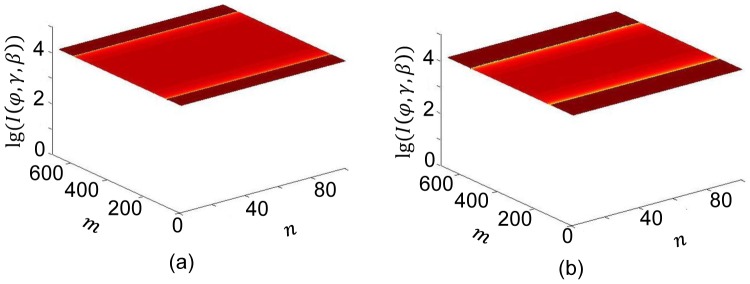
Numbers of detected photons along x-rays through the water phantom with the proposed dynamic bowtie (on a log scale). (a) and (b) Surface displays for the numbers of detected photons for 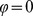 and 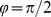 respectively.

FIG. 7 shows the numbers of detected photons 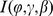 for 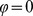 and 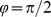 respectively. It is seen that the numbers of detected photons were made quite uniform with only slight variations mainly due to the container material for WB.

### Realistic Demonstration

**Figure 8:**
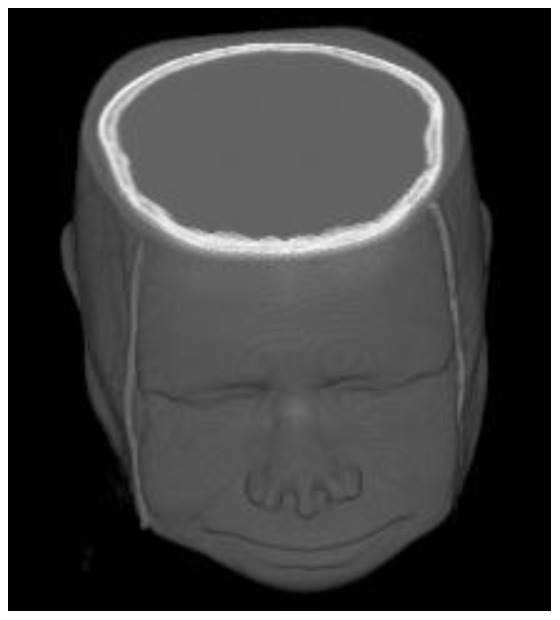
Head CT model approximated as an ellipse of semi-major axis 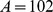 mm and semi-minor axis 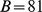 mm.

To show the practical value of our 3D dynamic bowtie, we selected a 3D CT image volume to simulate the detected photons and compared the dynamic ranges of the signals with and without the dynamic bowtie respectively. FIG. 8 is a 3D head CT volume from the Visible Human project [22]. After scaling, the head was approximated as a cylinder with an elliptical cross-section of a semi-major axis 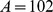 mm and a semi-minor axis 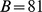 mm.

**Figure 9:**
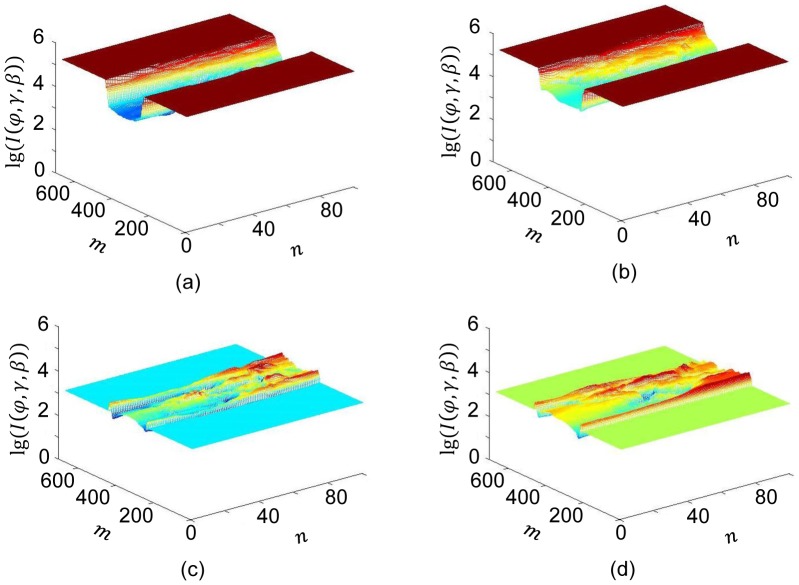
Numbers of detected photons along x-rays through the head CT volume without and with the proposed dynamic bowtie (on a log scale). (a) and (b) Numbers of detected photons assuming 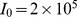 without a bowtie for 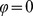 and 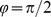 respectively. (c) and (d) Numbers of detected photons assuming 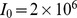 with a proposed bowtie for 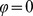 and 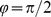 respectively.

As mentioned earlier, we designed a dynamic bowtie for the cylindrical water phantom of 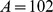 mm and 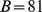 mm. FIG. 9 illustrates the numbers of detected photons for 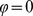 and 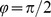 with and without the dynamic bowtie respectively. It is seen that the dynamic ranges of the signals differ greatly in the two cases, showing the advantage of the dynamic bowtie in FIG. 9(c) and (d).

## Discussion and Conclusions

As demonstrated in FIG. 7, for a water phantom it is feasible to make the expected numbers of detected photons almost the same across all the detector elements with a dynamic bowtie. If a cross section of a patient can be well approximated in this way, the dynamic range of detectors can be optimally matched to that of projection data. As shown in FIG. 9, without a bowtie only a small portion of the detector dynamic range is utilized to depict the signal variation. With a dynamic bowtie, the dynamic range problem can be basically remedied, leading to a reduced radiation dose for a given image quality requirement.

**Figure 10:**
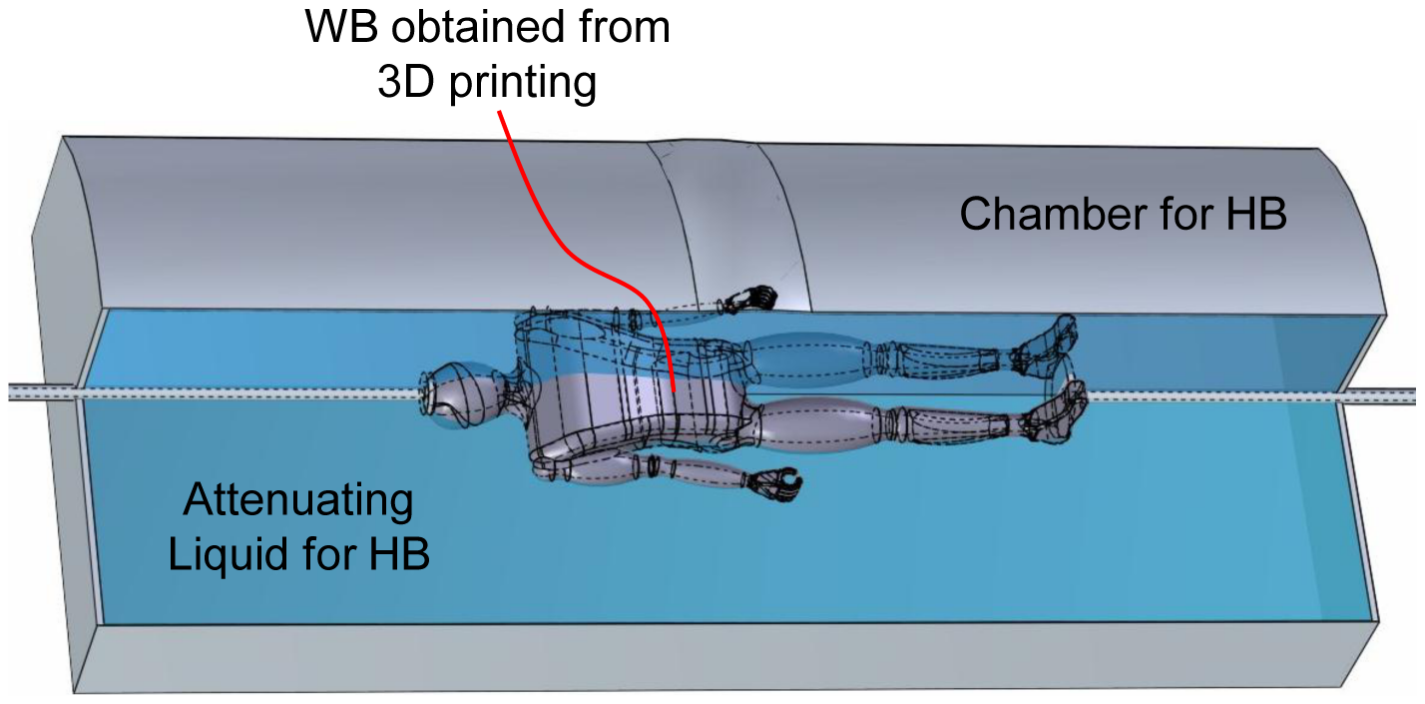
Dynamic bowtie for full body helical CT, in which the WB is rapidly prototyped according to an individualized patient contour obtained from surface scanning.

Our proposed approach for the design of a dynamic bowtie is to fit a rotating or spiraling WB into a liquid HB. For a clinical CT scan, it is desirable that the WB should be individualized before imaging. With the progress of the 3D surface scanning of human body [23], [24], and the popularization of 3D printing technology [25], [26], it seems feasible to individualize dynamic bowties via rapid prototyping based on an individualized optical surface model. Furthermore, this process can be completely automatic in the future, and finished in less than a couple of minutes. Specifically, we could just capture the body surface of a patient, deform a digital atlas such as the visible human dataset into the surface model, and produce rotating patient-specific WB. FIG. 10 illustrates a dynamic bowtie design for full-body helical cone-beam scanning. As aforementioned, the WB is a reduced copy of the patient. During helical cone-beam scanning, the WB movement will be fully synchronized with the helical scanning trajectory from the beginning to the end. It is underlined that the geometrical adaptability and technical feasibility of the proposed 3D dynamic bowtie promises significant performance improvement and dose saving.

In addition to the similarities and differences we have already discussed above between our proposed liquid-based device and the rigid solid beam shaper [17], here we would like to mention more unique merits associated with our approach. First, it is convenient to change the density of liquid HB by adjusting the solution concentration to adapt the device for different applications. Also, proper solution elements can be selected for various attenuating needs; for example, the cerium with the K-edge at 40 keV provides a beam well matched to the attenuation characteristics of iodine [18]. Besides the reusability of the liquid HB, our scheme is mechanically cheaper and easier to print a thin-walled plastic WB than the idea to print a hollow rigid solid. Finally, the WB is placed within the liquid HB as a single unit which is compact and efficient for system integration.

As explained above, the dynamic bowtie rotation and the helical scan are fully synchronized from the beginning to the end. In a typical CT system, a dynamic bowtie is a small part. Its control can be easily realized if the speed is not too rapid. When high speed is important for some applications, namely, cardiac imaging, we believe that our design should also work in principle but high precision engineering will be involved which could be expensive. In other words, other than cardiac imaging, the liquid bowtie design could be an interesting option.

It is acknowledged that in this initial study, a monochromatic x-ray source has been assumed. In practice, an x-ray source is polychromatic. The multi-energy spectrum introduces an additional layer of complexity. In this scenario, we need to match HB liquid and WB content with the x-ray spectrum being an additional key factor, augment the objective function in terms of numbers of detected photons and utilize the least square criterion for an overall optimization. In a follow-up study, we will perform the bowtie simulation for cone-beam and helical scan with a polychromatic spectrum, and analyze the dynamic range, noise and dose reduction with clinic datasets.

In conclusion, we have proposed a methodology for the design of dynamic bowties for multi-slice/cone-beam CT in either circular or helical scanning mode, and demonstrated its feasibility in a realistic numerical study. The main contribution is to embed a 3D WB as a miniature of a patient being scanned into a liquid HB, and coordinate their motions to compensate for any attenuation path differences. Further refinement of the current design is under way.
